# Engineering anti-Lewis-Y hu3S193 antibodies with improved therapeutic ratio for radioimmunotherapy of epithelial cancers

**DOI:** 10.1186/s13550-016-0180-0

**Published:** 2016-03-17

**Authors:** Ingrid J. G. Burvenich, Fook-Thean Lee, Graeme J. O’Keefe, Dahna Makris, Diana Cao, Sylvia Gong, Angela Rigopoulos, Laura C. Allan, Martin W. Brechbiel, Zhanqi Liu, Paul A. Ramsland, Andrew M. Scott

**Affiliations:** Tumour Targeting Laboratory, Ludwig Institute for Cancer Research and Olivia Newton-John Cancer Research Institute, Melbourne, VIC Australia; School of Cancer Medicine, La Trobe University, Melbourne, VIC Australia; Department of Molecular Imaging and Therapy, Austin Health, Melbourne, Australia; Radioimmune and Inorganic Chemistry Section, Radiation Oncology Branch, Center for Cancer Research, National Cancer Institute, Bethesda, MD USA; School of Science, RMIT University, Bundoora, VIC Australia; Centre for Biomedical Research, Burnet Institute, Melbourne, VIC Australia; Department of Immunology, Monash University, Melbourne, VIC Australia; Department of Surgery Austin Health, University of Melbourne, Heidelberg, VIC Australia; Faculty of Medicine, University of Melbourne, Melbourne, VIC Australia; Olivia Newton-John Cancer Research Institute, 145-163 Studley Road, Heidelberg, VIC 3084 Australia

**Keywords:** Therapeutic ratio, Payload delivery, Antibody engineering, Small animal imaging, Lewis-Y

## Abstract

**Background:**

The aim of the study was to explore Fc mutations of a humanised anti-Lewis-Y antibody (IgG1) hu3S193 as a strategy to improve therapeutic ratios for therapeutic payload delivery.

**Methods:**

Four hu3S193 variants (I253A, H310A, H435A and I253A/H310A) were generated via site-directed mutagenesis and radiolabelled with diagnostic isotopes iodine-125 or indium-111. Biodistribution studies in Lewis-Y-positive tumour-bearing mice were used to calculate the dose in tumours and organs for therapeutic isotopes (iodine-131, yttrium-90 and lutetium-177).

**Results:**

^111^In-labelled I253A and H435A showed similar slow kinetics (*t*_1/2β_, 63.2 and 62.2 h, respectively) and a maximum tumour uptake of 33.11 ± 4.05 and 33.69 ± 3.77 percentage injected dose per gramme (%ID/g), respectively. ^111^In-labelled I253A/H310A cleared fastest (*t*_1/2β_, 9.1 h) with the lowest maximum tumour uptake (23.72 ± 0.85 %ID/g). The highest increase in tumour-to-blood area under the curve (AUC) ratio was observed with the metal-labelled mutants (^90^Y and ^177^Lu). ^177^Lu-CHX-A" DTPA-hu3S193 I253A/H310A (6:1) showed the highest tumour-to-blood AUC ratio compared to wild type (3:1) and other variants and doubling of calculated dose to tumour based on red marrow dose constraints.

**Conclusions:**

These results suggest that hu3S193 Fc can be engineered with improved therapeutic ratios for ^90^Y- and ^177^Lu-based therapy, with the best candidate being hu3S193 I253A/H310A for ^177^Lu-based therapy.

**Electronic supplementary material:**

The online version of this article (doi:10.1186/s13550-016-0180-0) contains supplementary material, which is available to authorized users.

## Background

The use of monoclonal antibodies (mAbs) to deliver radioisotopes to tumour sites (radioimmunotherapy (RIT)) has been very successful in the treatment of haematologic tumours as the targeted radiation is able to kill the highly radiosensitive leukemias and lymphomas. Treatment of solid tumours that are generally more radioresistant has been less successful due to several factors including long serum persistence, poor tumour penetration and slow diffusion rates of mAbs [1]. The large molecular size of mAbs (150 kDa) does not allow renal excretion. In addition, the Fc fragment of IgG antibodies interacts with the neonatal Fc receptor (FcRn, Brambell receptor), preventing it from being degraded in the lysosomes [2–6]. The resulting long serum persistence of large-sized mAbs cause dose-limiting radiotoxicity to normal tissues, principally red marrow.

The choice of the optimal radionuclide for RIT depends on its intended use. Physical properties such as path length, emission energy and physical half-life must correlate with a particular tumour size. ^131^I (*E*_max_, 0.66 MeV), suitable for tumours <1 cm, and ^90^Y (*E*_max_, 2.3 MeV), suitable for tumours >1 cm, are both β-particle-emitting isotopes and have been used in >95 % of clinical RIT trials [1]. Similar to ^131^I, ^177^Lu (*E*_max_, 0.5 MeV) has been identified as having favourable characteristics for treatment of tumours particularly with heterogeneous antigen expression [7]. Whilst ^90^Y and ^177^Lu labels are retained intracellularly after endocytosis, the ^131^I label is rapidly released from the cells [8, 9].

Early engineering approaches to reduce the serum half-life of antibodies and improve therapeutic ratios were based on reduction in antibody size by deletion of constant domains [1, 10, 11]. Our laboratory has previously evaluated smaller forms such as diabody, F(ab′)_2_ and tetrameric scFv constructs without an Fc fragment. These constructs showed a rapid elimination phase compared to wild-type IgG1, more rapid tumour targeting with the maximum tumour uptake observed at an earlier time point but strongly reduced maximal tumour uptake [12–14]. Another approach studied in preclinical studies to improve therapeutic ratio is pretargeted RIT. The multistep targeting approach allows the antibody to clear first before administrating the therapeutic radionuclide, and this approach has shown high increases in therapeutic ratio [1, 15].

The FcRn receptor recycles IgG antibodies into the blood circulation. During the last decade, the Fc-FcRn binding site has been well characterised, with I253, H310 in the CH2 domain and H435 in the CH3 domain identified as the key residues involved in the Fc-FcRn binding [16–19]. The FcRn-mediated IgG recycling can be described as a three-step process: (1) passive pinocytosis of IgG into endothelial cells; (2) in the acidic environment of endosomes (pH <6.5), histidine residues in the Fc fragment of IgG become protonated and this allows high-affinity binding of IgG to FcRn; and (3) unbound IgG will undergo lysosomal degradation whilst FcRn-bound IgG is released back into the blood circulation [19]. Therefore, more recent engineering approaches to reduce the serum half-life of antibodies or Fc-coupled biological compounds used for payload delivery focus on mutating the specific amino acids in the CH2 or CH3 domain of the Fc fragment involved in the binding site of FcRn. Recycling of the variants is reduced, due to their diminished interaction with the FcRn receptor at pH <6.5. Most work has been done with radiolabelled Fc-containing antibody fragments such as scFv-Fc [20–23] and a minibody [22]. Only one intact radiolabelled antibody (chTNT-3) carrying the I253A mutation has been studied in biodistribution studies and imaging studies [24].

We recently produced anti-Lewis-Y (Le^y^) humanised IgG1 variants carrying specific mutations in the Fc-FcRn binding region and investigated their binding properties to murine FcRn (muFcRn) and human FcRn (huFcRn) in vitro and in vivo [25]. A good correlation was found with low in vitro binding to FcRn corresponding to fast blood clearance rates. Differences in blood clearance rates were observed between the murine and human FcRn mouse model, showing that one alanine mutation in the Fc-huFcRn binding site (i.e. I253A, H310A or H435A) was sufficient to completely abrogate binding to huFcRn and generate hu3S193 antibodies with clearance rates as fast as hu3S193 antibody fragments (e.g. (Fab′)_2_) without an Fc fragment. In contrast, two alanine mutations in hu3S193 Fc (e.g. I253A/H310A) were necessary to completely abrogate binding to muFcRn. Single non-alanine variants of hu3S193 that were able to completely abrogate muFcRn binding were I253D, I253P, H310D and H310E [25].

This study explored the impact of amino acid substitutions on conserved residues of hu3S193 that have been shown to be critical for maintaining serum persistence of human IgG1 antibodies as a means to improve therapeutic ratios for RIT in solid tumours using intact antibodies. Hu3S193 has been shown to have significant anti-tumour effect in animal models for delivery of radioisotopes, including ^131^I, ^90^Y and ^177^Lu [26–29]. A first-in-man phase I trial of hu3S193 in patients with Le^y^ expressing tumours clearly showed the potential for delivery of radioisotopes using hu3S193 for selective targeting of solid tumours [31]. The utility of hu3S193 mutants in cancer diagnosis and therapy were evaluated by pharmacokinetic, biodistribution, dosimetry and immunoscintigraphy studies in tumour-bearing mice.

## Methods

### Construction of hu3S193 mutant antibodies

The construction and production of hu3S193 and huA33 has been described before [31, 32]. The hu3S193 heavy chain (HC) was ligated into the pEE6.4 mammalian expression vector (Lonza Biologics, Slough, UK) via a *Hin*dIII and *Eco*RI double digest (pEE6.4/hu3S193 HC). pEE6.4/hu3S193 HC was used as a template for site-directed mutagenesis (GeneTailor™ Site-Directed Mutagenesis System (Invitrogen) or QuickChange II XL Site-Directed Mutagenesis Kit (Stratagene) to introduce the following substitutions in the CH2 and CH3 domains: I253A, H310A, H435A and I253A/H310A [25]. The hu3S193 kappa light chain was ligated into the pEE14.4 mammalian expression vector containing the glutamine synthetase gene (Lonza Biologics) via a *Hin*dIII and *Eco*RI double digest. For the simultaneous expression of each mutated hu3S193 antibody, light and mutated heavy chain genes were cloned into a double-gene vector (pDGV) using a *Not*I and *Pvu*I double digest. Freestyle 293-F cells (1 × 10^6^ cells/mL) were transfected with the pDGV constructs according to the manufacturer’s instructions (Invitrogen). Supernatants were harvested at 96 h post transfection. Hu3S193 antibodies were purified using HiTrap KappaSelect columns (GE Healthcare). Purified proteins were analysed by SDS-PAGE under non-reducing conditions and evaluated by size exclusion chromatography on a Superdex 200 HR 10/30 column (GE Healthcare) using 0.01 mol/L sodium phosphate and 0.15 mol/L NaCl (pH 7.2) as elution buffer.

### In vitro binding to Lewis-Y and protein A of hu3S193 mutant antibodies

The Lewis-Y binding activity of hu3S193 antibodies was determined by BIAcore analysis using a synthetic Lewis-Y tetrasaccharide coupled to BSA (Alberta Research Council, Edmonton, Alberta, Canada) on a CM5 chip using a BIAcore 2000 as described (BIAcore AB, Uppsala, Sweden) [30]. Hu3S193 antibody samples were diluted in HBS buffer (10 mM HEPES, pH 7.4, 150 mM NaCl, 3.4 mM di-Na-EDTA and 0.005 % Tween 20). Aliquots (60 μL) were injected over the sensor chip surface at a flow rate of 30 μL/min. After the injection phase, dissociation was monitored by flowing HBS over the chip surface for 300 s. Bound antibody was eluted, and the chip surface was regenerated between samples by injection of 100 mM HCl. For kinetic analysis, varying concentrations of hu3S193 wild type and mutants were injected (10, 19, 38, 75 and 150 nmol/L). Global analysis using a 1:1 Langmuir model fit was performed using BIA-evaluation version 4.1.1 software.

Fluorescence-activated cell sorter (FACS) analysis was done on Lewis-Y-positive A431 skin cancer cells. Aliquots of 2 × 10^5^ cells were incubated with hu3S193 antibodies (200 nmol/L) in DMEM/F12 medium containing 10 % foetal bovine serum on ice. After washing the cells with PBS, cells were incubated with a goat phycoerythrin-conjugated anti-human IgG (Sigma) and incubated on ice for 30 min. Cells were washed with phosphate-buffered saline and resuspended in a final volume of 300 μL. In control samples, primary antibody was omitted. Flow cytometric analysis was done using a Guava EasyCyte Plus flow cytometer. Cancer cell populations were gated based on forward and side scatter variables. Data analysis was done using WinMDI (Joseph Trotter).

### Radiolabelling of hu3S193 antibodies

Hu3S193 antibodies were radiolabelled with four isotopes: ^125^I, ^131^I, ^111^In and ^177^Lu. Iodine-125 and lutetium-177 were obtained from PerkinElmer (PerkinElmer Life and Analytical Sciences, Waltham, MA), iodine-131 was obtained from ANSTO (ANSTO, Menai, Australia) and indium-111 was obtained from MDS Nordion (Canada).

Radioiodination was performed using pH-neutralised isotopes, catalysed by iodogen-coated glass beads as previously published [12, 33]. After a 10-min incubation period, the reaction mixture was purified through a Sephadex G50 desalting column (Sigma-Aldrich, Sydney, Australia) equilibrated with 0.9 % NaCl containing 0.05 % human serum albumin.

Radiolabelling of the hu3S193 antibody constructs with indium-111 and lutetium-177 was done by using the bifunctional metal ion chelate C-functionalized *trans*-cyclohexyl diethylenetriaminepentaacetic acid (CHX-A″ DTPA) [34, 35]. A chelate to antibody ratio of 3:1 was employed, using 0.1 M sodium bicarbonate buffer (pH 8.6) containing 0.9 % NaCl. Incubation was allowed for 16 h at room temperature. Under these conditions, one to two chelates are expected per antibody molecule. The radiolabelled mixture was purified through a Sephadex G50 desalting column equilibrated with 0.9 % NaCl containing 0.05 % human serum albumin.

Radiolabelling was performed on the day of injection into mice. Prior to injection, the percentage of unbound radionuclide content was determined by ITLC as previously described [36]. Determination of the immunoreactivity of radiolabelled hu3S193 antibody constructs was performed by a single-point binding assay, where 10 × 10^6^ Le^y^-positive A431 cells were incubated with 20 ng of radiolabelled antibody constructs for 45 min at room temperature with continuous mixing throughout to keep the cells in suspension. Cells were washed three times, and pellets were measured in a gamma counter (Cobra II, Model 5002, Packard Instruments, Canberra, Australia). Three samples of radiolabelled antibody at the same concentration as that initially added to the cells were measured at the same time of the cell pellets, and immunoreactivity was calculated: (cpm cell pellet/mean cpm radioactive antibody standards) × 100. Serum stability was analysed by determination of immunoreactivity on the day of injection, at 48 h, and 7 days after 20 ng of radiolabelled antibody was incubated in human serum at 37 °C.

### Blood clearance studies

Female athymic mice (BALB/c *nu*/*nu*; 4–6 weeks; Animal Resources Centre) were injected in the tail vein with 0.185 MBq ^125^I-mutant (2.5–5 μg, 5 μCi) and 0.185 MBq ^131^I-hu3S193 (2.5–5 μg, 5 μCi) wild type antibody (*n* = 5). A separate group of athymic mice were injected with 0.185 MBq ^125^I- hu3S193 mutant (2.5–5 μg, 5 μCi) and 0.185 MBq ^111^In-hu3S193 (2.5–5 μg, 5 μCi) mutant (*n* = 5). All animal studies were approved by the Austin Hospital Animal Ethics Committee and were conducted in compliance with NHMRC Australian code of practice for the care and use of animals for scientific purposes. Blood samples (10–20 μL) were collected from groups of five at 5 min, 1, 2, 4, 8, 24, 48, 72, 120, 168, 240 and 336 h after injection of radioactive antibodies. Samples were counted in a gamma counter (Cobra II). Standards prepared from injected material were counted each time with blood samples enabling calculations to be corrected for physical decay of isotope.

### Biodistribution studies

Biodistribution studies were done with athymic non-tumour-bearing mice (BALB/c *nu*/*nu*, female, 4–6 weeks) or mice bearing A431 tumours (^111^In, 0.657 ± 0.216 g; ^131^I, 0.515 ± 0.195 g; ^177^Lu, 0.466 ± 0.086 g). To study the tumour uptake and biodistribution in normal tissues of radioiodinated hu3S193 variants, A431 tumour-bearing BALB/c *nu*/*nu* mice were co-injected intravenously with 0.185–0.74 MBq (5–20 μCi) ^131^I-hu3S193 variants (2–6 μg protein) and 0.185–0.74 MBq ^125^I-hu3S193 wild type (2–6 μg protein). In a second study, 0.185–0.74 MBq ^111^In-CHX-A″ DTPA-labelled mutant (2–6 μg protein) and 0.185-0.74 MBq ^125^I-labelled hu3S193 wild type (2–6 μg protein) were co-injected. ^111^In-CHX-A″ DTPA-labelled wild type and ^125^I-hu3S193 wild type were also injected as a control group. ^125^I-hu3S193 wild type was used as an internal control in all injected animals to enable direct comparison between the different mutants. Typically, groups of four to five mice were sacrificed at 4, 24, 48, 72, 120, 168, and 240 or 288 h after injection of radiolabelled antibodies. For evaluation of ^177^Lu-labelled antibodies, mice were sacrificed at 48 h after injection. At the designated time points, groups of mice (*n* = 4–5) were humanely sacrificed by over-inhalation of isoflurane. Mice were bled by cardiac puncture, and tumours and organs (skin, liver, spleen, small intestine, stomach, kidney, brain, bone (femur), lungs and heart) were immediately removed and blotted dry. All samples were weighed and counted in a dual gamma scintillation counter (Cobra II, Packard Instruments). Triplicate standards prepared from the injected material were counted at each time point with tissue and tumour samples enabling calculations to be corrected for the physical decay of the isotopes. Results of the radiolabelled antibody distribution over time were calculated as the mean percentage injected dose per gramme (%ID/g ± SD) for each mutant and parental hu3S193 per time point.

### Pharmacokinetic analysis and predictive dosimetry

Antibody serum concentrations were expressed as percentage injected dose per millilitre (%ID/mL), and the blood concentrations (μg/mL) were calculated. A two-compartment IV bolus model with macro-parameters, no lag time and first order elimination (WinNonlin Model 8) was fitted to serum data obtained from blood clearance studies for each animal using unweighted non-linear, least squares with WinNonLin version 5.2 (Pharsight Corp., Mountain View, CA). Estimates were determined for the pharmacokinetic parameters: alpha half-life (*t*_1/2α_), beta half-life (*t*_1/2β_), area under the curve extrapolated to infinity (AUC_0-∞_) and mean residence time (MRT). Alpha half-lives of mutant hu3S193 were constrained to be smaller or equal to the alpha half-life of hu3S193 wild type. Significant differences in these values were examined by comparing the coefficient of variation (CV %) for the estimated parameters.

To estimate radioimmunotherapeutic applications for ^90^Y- or ^177^Lu-labelled mutants, biodistribution data obtained with ^111^In-CHX-A″ DTPA-labelled antibodies were used to generate time-activity curves for the calculations of predictive radiation doses for the bone marrow, liver, kidneys and tumour. Identical biodistribution and biologic clearance of ^111^In-, ^90^Y- and ^177^Lu-labelled antibodies were assumed. Predictive dosimetric analysis was also done for ^131^I-labelled antibodies based on biodistribution data obtained with ^125^I-labelled antibodies. As radiation absorbed doses are proportional to %ID/g, time-activity curves for the blood, liver, kidneys and tumour were integrated over time to calculate area under the curve (AUC). Time-activity curves generated from biodistribution data were corrected for radiodecay. Therefore, the pharmacokinetic values calculated from such data refer to pharmacological values of the antibodies in the absence of radioisotope. Appropriate physical half-life corrections were applied to convert %ID_pharmacological_/g to %ID_radioisotope_/g, and the time-activity curves were fit to either a two or three exponential function from which the AUC is determined for ^90^Y, ^177^Lu and ^131^I. AUC integration from zero to infinity was done by the sum of a trapezoidal integration of the measurement range (AUC_0–288 h_) plus an extrapolated model fit for the extrapolated range (AUC_288 h–∞_). To calculate accumulated activity, photon dose and edge effects were ignored. Dose to red marrow was determined from blood concentrations using a baseline value of 0.1 [21].

### Nano-SPECT and MR imaging

All the SPECT and MR scans were performed on a small animal nano-SPECT/CT imaging system and a small animal nano-PET/MR imaging system (Mediso nano-Scan^PM^, Mediso Medical Imaging Systems, Budapest, Hungary) individually. Groups of two mice were injected with 3.7 MBq (270 μg) ^177^Lu-CHX-A″ DTPA-labelled antibody (huA33 wild type, hu3S193 wild type or hu3S193 I253A/H310) and serially imaged at 2 days post injection. Imaging procedures involved anaesthesia of mice by isoflurane. Each mouse was scanned in supine position with its head secured via ear and tooth bars. Respiration was monitored by a pressure-sensitive pad adhered to the abdomen. The imaging study started with a whole body T1-weighted MR scan, followed by a 60-min SPECT scan which was reconstructed into a volumetric image with the voxel size of 0.3 mm × 0.3 mm × 0.3 mm using the Mediso Tera-TOMO® Monte Carlo-based algorithm. Subsequently, reconstructed SPECT and MR images were transferred to a research PACS system where the images could be retrieved for further processing and analysis.

To confirm data obtained from imaging, groups of five mice bearing A431 tumours were injected with 6.29 MBq (100 μg) ^177^Lu-CHX-A″ DTPA-labelled antibody (huA33 wild type, hu3S193 wild type or hu3S193 I253A/H310) and sacrificed at 48 h post injection. Organs and blood were collected as described before, and the radiolabelled antibody distribution over time was calculated as the mean %ID/g ± SD for each antibody per time point.

### Statistical analysis

To compare tumour uptake at different time points for the mutants versus hu3S193 wild type, two-way ANOVA with the Bonferroni post test was performed using GraphPad Prism version 5.00 for Windows, GraphPad Software, San Diego California USA www.graphpad.com. Tumour-to-tissue ratios or tissue uptake at specific time points were analysed using one-way ANOVA with Tukey’s multiple comparison post test. When less than three groups were compared, a non-parametric *t* test (one-tailed) was used.

## Results

### Production of hu3S193 variants with short elimination half-lives

To investigate whether hu3S193 antibodies with reduced serum persistence would benefit payload delivery, three hu3S193 single variants (I253A, H310A, H435A) and one double variant (I253A/H310A) were generated. All antibodies were expressed in transiently transfected freestyle 293-F cells. Expression yields of antibodies ranged from 15 to 40 mg/L in shake flasks containing 60 mL medium. HiTrap KappaSelect columns were used to purify antibodies. Quality control using SDS-PAGE and HPLC on a Superdex 200 column showed highly pure antibody preparations with >98 % purity. All mutants maintained effective antigen binding in FACS analysis and BIAcore (Additional file 1: Table S1).

### Radiolabelling of hu3S193 constructs and huA33 control

Antibodies were radiolabelled with iodine-125 and indium-111, and radiochemical purity of all injected antibodies was more than 98 %. Immunoreactivity was determined in the presence of human serum at 37 °C for up to 6 days of incubation, as measured by percentage of antibody binding to Lewis-Y-positive A431 cells in a single-point immunoreactivity assay. Data presented in Table 1 demonstrates minimal loss in binding of all variants compared to wild type at day 0 due to labelling (20–35 %). Loss of immunoreactivity due to incubation in human serum at 37 °C was similar for variants and wild type (day 7: 55–70 %).

**Table 1:** Immunoreactive fraction (% total binding) of hu3S193 antibodies incubated in human serum at 37 °C for 6 days

Radiolabel	Antibody	D0	D2	D6
^125^I-	Wild type	78.64 ± 1.59^a^	59.09 ± 5.54	32.36 ± 0.12
	I253A	66.76 ± 0.33	47.50 ± 2.69	30.99 ± 0.77
	H310A	70.75 ± 3.99	53.52 ± 3.53	32.95 ± 0.28
	H435A	69.50 ± 0.33	54.59 ± 4.68	34.82 ± 2.98
	I253A/H310A	71.93 ± 3.22	58.29 ± 0.33	30.52 ± 1.45
^111^In-	Wild type	86.84 ± 0.74	64.46 ± 8.04	40.21 ± 0.62
	I253A	83.47 ± 4.26	59.16 ± 3.05	40.77 ± 0.53
	H310A	81.03 ± 3.38	55.83 ± 1.78	35.64 ± 0.29
	H435A	83.38 ± 3.81	59.70 ± 7.27	46.19 ± 4.63
	I253A/H310A	86.07 ± 2.74	57.18 ± 11.61	42.54 ± 1.15

^a^Data are presented as average ± SD (D2 ^125^I-, *n* = 2; D6 ^125^I- and ^111^In-, *n* = 2; rest *n* = 4)

Blood clearance studies were performed in BALB/c *nu*/*nu* mice (*n* = 5) using ^125^I-labelled antibody co-injected with their ^111^In-CHX-A″ DTPA-labelled counterpart, and blood clearance parameters were calculated (Table 2). There was no significant difference between the elimination half-lives (*t*_1/2β_) of radioiodinated or radiometal-chelated hu3S193 wild type. Radioiodinated hu3S193 variants showed shorter half-lives than their radiometal counterpart; the shorter the half-life the smaller the differences in half-lives observed between radioiodinated and radiometal-chelated hu3S193 mutants. Although different terminal serum half-lives were seen dependent on the choice of isotope, a similar ranking order of area under the curve (AUC) was observed: wild type>H435A = I253A>H310A>I253A/H310A (Table 2).

**Table 2:** Blood clearance parameters for ^125^I- and ^111^In-CHX-A″ DTPA-labelled hu3S193 antibodies

Radiolabel	Antibody	*t*_1/2α_ (h)	A_α_^a^ (%ID/g)	*t*_1/2β_ (h)	A_β_ (%ID/g)	AUC^b^ (%ID/g h)	MRT^c^ (h)
^125^I-	Wild type	2.5	36.5	140.7	35.3	7304	199.4
	I253A	0.7	18.1	13.2	60.0	1161	18.7
	H310A	0.6	17.7	7.6	74.0	824	10.7
	H435A	1.2	28.8	15.6	56.2	1316	21.7
	I253A/H310A	0.5	15.7	7.3	66.5	710	10.3
^111^In-CHX-A″ DTPA-	Wild type	2.6	37.2	151.5	31.5	7033	214.4
	I253A	2.7	34.1	63.2	44.6	4200	88.46
	H310A	1.5	38.9	24.4	46.9	1732	33.6
	H435A	1.9	39.6	62.2	38.6	3567	87.0
	I253A/H310A	0.5	20.0	9.1	58.7	781	12.9

^a^Amplitudes of the two components *t*_1/2α_ and *t*_1/2β_ are given by A_α_ and A_β_, respectively, where the sum of A_α_ and A_β_ is the total %ID/g

^b^ *AUC* area under the curve; time integral of the blood uptake

^c^ *MRT* mean residence time; used to give a single variable for blood clearance

### Biodistribution studies in tumour-bearing mice with ^131^I- and ^111^In-CHX-A″ DTPA-labelled hu3S193 antibodies

**Fig. 1:**
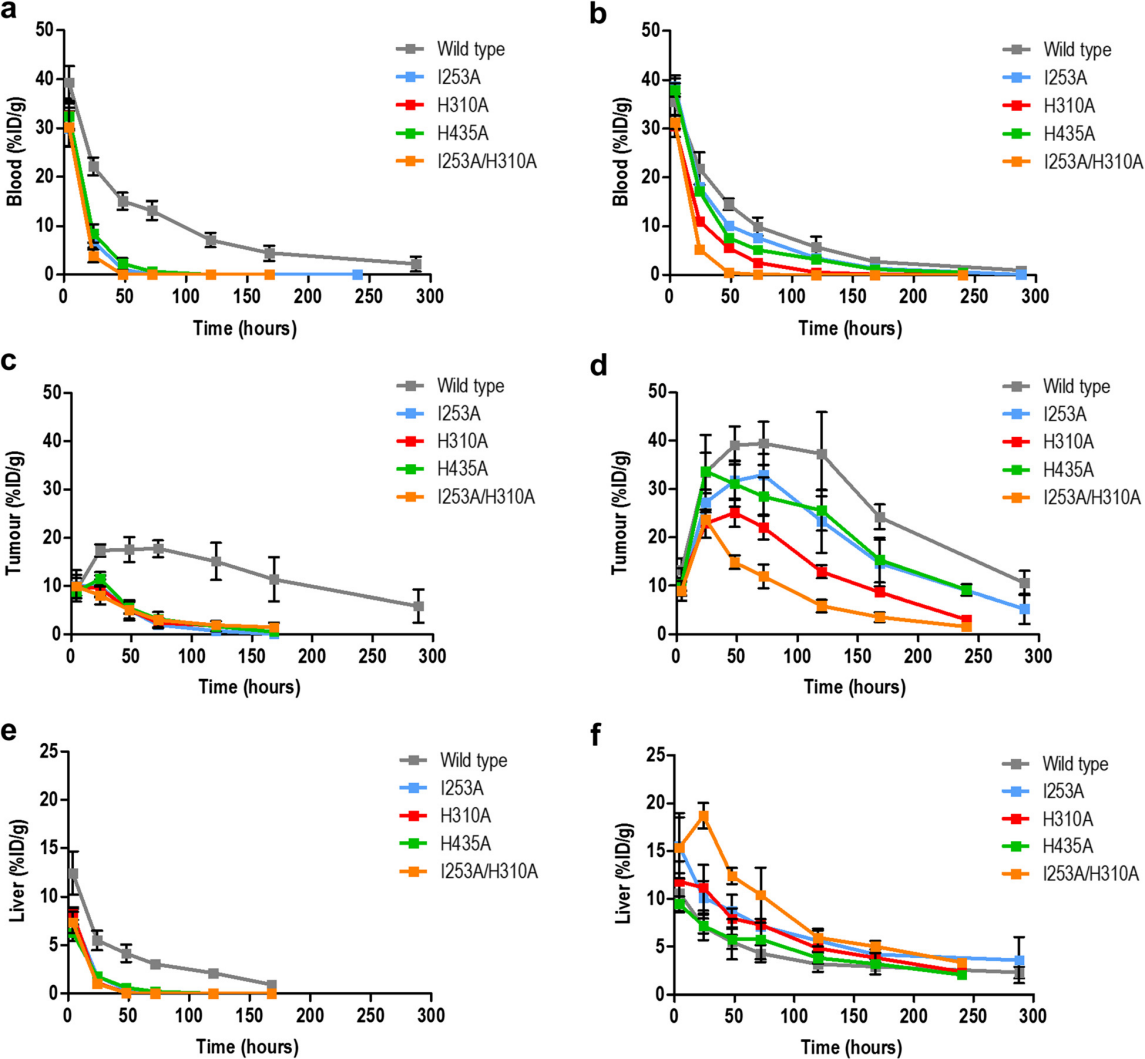
Biodistribution with ^**131**^I- (**a**, **c** and **e**) and ^**111**^In-CHX-A″ DTPA-labelled (**b**, **d** and **f**) hu3S193 antibodies. *n* = 5; bars, SD

In general, a significant reduction in tumour uptake was observed with faster clearing variants; the faster the blood clearance of the variant, the higher the reduction in tumour uptake (Fig. 1). Additional file 1: Table S2 shows the biodistribution results of ^111^In-CHX-A″ DTPA-labelled antibodies. Additional file 1: Table S3 shows the biodistribution results of ^131^I-labelled antibodies. Two-way ANOVA of differences in tumour uptake at different time points post injection of each variant compared to wild type is shown in Additional file 1 (^111^In-labelled variants versus ^111^In-labelled wild type, Additional file 1: Figure S1; ^131^I-labelled variants versus ^125^I-labelled wild type, Additional file 1: Figure S2). Radioiodinated hu3S193 antibodies cleared faster than their ^111^In-chelated counterparts, and as a result, lower tumour uptake was observed with radioiodinated variants compared to their ^111^In-chelated counterparts (Fig. 1, Additional file 1: Table S2 and Additional file 1: Table S3).

Although a significant reduction in tumour uptake was observed with faster clearing hu3S193 mutants, more favourable tumour-to-blood ratios were observed for ^111^In-labelled hu3S193 H310A and ^111^In-labelled hu3S193 I253A/H310A compared to wild type (Table 3). At 48 h post injection, ^111^In-labelled wild type showed a tumour-to-blood ratio of 2.70 ± 0.26 compared to 5.04 ± 2.04 for H310A (*P* < 0.05) and 32.81 ± 7.10 for I253A/H310A (*P* < 0.001). Tumour uptake was significantly lower for all ^131^I-labelled mutants compared to wild type (*P* < 0.0001) at all time points except 4 h post injection (Fig. 1, Table 3, Additional file 1: Table S3, Additional file 1: Figure S2). No significant differences in tumour uptake were observed between mutants. As seen with the ^111^In-labelled mutants at 48 h post injection, tumour-to-blood ratios for ^131^I-labelled mutants were more favourable than wild type (Table 3).

**Table 3:** Tumour uptake, blood levels and tumour-to-blood ratios for ^131^I- and ^111^In-CHX-A″ DTPA-labelled hu3S193 antibody variants in A431 tumour-bearing mice

Antibody	Time (hours)	Tumour (%ID/g)	Blood (%ID/g)	T/B	Tumour (%ID/g)	Blood (%ID/g)	T/B
		^131^I			^111^In		
Wild type	4	9.02 ± 0.73^a^	39.37 ± 3.33	1:4	13.17 ± 2.40	35.56 ± 5.48	1:3
	24	17.37 ± 1.25	22.17 ± 1.81	1:1	33.42 ± 7.75	21.83 ± 3.32	2:1
	48	17.58 ± 2.60	15.09 ± 1.70	1:1	39.01 ± 3.92	14.50 ± 1.11	3:1
	72	17.76 ± 1.72	13.18 ± 1.91	1:1	39.27 ± 4.33	9.97 ± 1.85	4:1
	120	15.10 ± 3.89	7.13 ± 1.46	2:1	37.26 ± 8.67	5.78 ± 2.12	6:1
	168	11.40 ± 4.56	4.44 ± 1.56	2:1	24.24 ± 2.57	1.06 ± 0.32	9:1
I253A	4	10.01 ± 3.25	29.95 ± 3.59	1:3	9.64 ± 0.74	38.74 ± 3.57	1:4
	24	9.95 ± 2.26	6.94 ± 1.34	1:1	27.17 ± 2.00	17.99 ± 1.57	2:1
	48	5.07 ± 2.10	1.09 ± 0.32	5:1	31.75 ± 3.98	10.19 ± 1.04	3:1
	72	2.03 ± 0.81	0.04 ± 0.08	48:1	33.11 ± 4.05	7.64 ± 1.32	4:1
	120	0.78 ± 0.79	0.00 ± 0.00	>500:1	23.68 ± 6.52	2.99 ± 0.73	8:1
	168	0.10 ± 0.14	0.00 ± 0.00	>500:1	14.66 ± 4.80	1.14 ± 0.63	13:1
H435A	4	8.79 ± 0.45	32.46 ± 2.63	1:4	10.25 ± 1.30	37.97 ± 2.68	1:4
	24	11.52 ± 1.38	8.46 ± 1.89	1:1	33.69 ± 3.77	17.09 ± 1.59	2:1
	48	5.53 ± 1.18	2.27 ± 1.21	2:1	31.10 ± 4.76	7.65 ± 1.60	4:1
	72	3.18 ± 0.22	0.69 ± 0.17	5:1	28.48 ± 4.00	5.18 ± 1.40	6:1
	120	1.64 ± 1.09	0.00 ± 0.00	>500:1	25.60 ± 4.16	3.19 ± 0.62	8:1
	168	0.54 ± 0.58	0.00 ± 0.00	>500:1	15.34 ± 4.65	1.16 ± 0.62	13:1
H310A	4	9.80 ± 1.83	32.56 ± 3.00	1:3	10.79 ± 2.87	30.52 ± 5.06	1:3
	24	9.58 ± 1.73	3.85 ± 0.35	3:1	22.85 ± 2.93	11.05 ± 1.62	2:1
	48	5.10 ± 1.87	0.01 ± 0.02	>500:1	25.10 ± 2.91	5.61 ± 2.01	5:1
	72	2.43 ± 1.10	0.00 ± 0.00	>500:1	22.12 ± 2.55	2.56 ± 0.60	9:1
	120	1.81 ± 0.71	0.00 ± 0.00	>500:1	12.89 ± 1.33	0.54 ± 0.16	24:1
	168	1.45 ± 0.99	0.00 ± 0.00	>500:1	8.64 ± 0.96	0.17 ± 0.06	51:1
I253A/H310A	4	9.90 ± 2.42	30.20 ± 3.97	1:3	8.92 ± 2.00	28.84 ± 6.05	1:3
	24	8.04 ± 1.82	3.88 ± 1.32	2:1	23.72 ± 0.85	5.28 ± 0.81	5:1
	48	5.10 ± 0.76	0.06 ± 0.09	80:1	14.84 ± 1.43	0.46 ± 0.07	32:1
	72	3.04 ± 1.58	0.00 ± 0.00	>500:1	11.92 ± 2.46	0.08 ± 0.03	154:1
	120	1.92 ± 0.53	0.00 ± 0.00	>500:1	5.85 ± 1.31	0.00 ± 0.00	>500:1
	168	1.43 ± 0.27	0.00 ± 0.00	>500:1	3.52 ± 1.00	0.00 ± 0.00	>500:1

^a^Data presented as percentage injected dose per gramme tissue (mean ± SD, *n* = 5)

As expected for large proteins (>60 kDa), all hu3S193 variants exhibited a hepatic clearance. This is evidenced by the ^111^In-labelled variants showing an increase in liver uptake at early time points with increased blood clearance rates (Fig. 1f). Liver uptake for the slowest clearing mutants hu3S193 H435A and hu3S193 I253A peaked at 4 h p.i. (9.49 ± 0.76 %ID/g and 15.52 ± 3.45 %ID/g, respectively). The fastest mutant hu3S193 I253A/H310A reached a maximum liver uptake at 24 h p.i. (18.68 ± 1.34 %ID/g).

### Improved therapeutic ratio with Fc-engineered hu3S193 mutants

To calculate the therapeutic ratio for hu3S193 variants with faster blood clearance, AUC values were calculated for blood of labelled (^131^I-, ^90^Y-, ^177^Lu-) antibodies and tumour-to-blood AUC ratios were calculated (Table 4). With exception of the I253A variant, all mutants demonstrated higher or similar tumour-to-blood AUC ratios compared to hu3S193 wild type. The highest increase in tumour-to-blood AUC ratio was observed with the metal-labelled mutants with ^177^Lu-CHX-A″ DTPA-hu3S193 I253A/H310A showing a doubling in the tumour-to-blood AUC ratio compared to hu3S193 wild type and other variants. No increase in tumour-to-blood AUC ratios was observed with the faster clearing radioiodinated variants.

**Table 4:** AUC values (%ID/g h) and therapeutic ratios of ^131^I-, ^90^Y-CHX-A″ DTPA- and ^177^Lu-CHX-A″ DTPA-labelled hu3S193 antibody variants in A431 tumour-bearing mice

Radiolabel	Antibody	AUC_tumour_	AUC_blood_	AUC_liver_	AUC_kidney_	T/L^a^	T/K^b^	T/B^c^
^90^Y-CHX-A" DTPA-	Wild type	2849	1276	498	636	5.7	4.5	2.2
	H435A	2168	964	514	561	4.2	3.9	2.3
	I253A	1541	1126	738	633	2.1	2.4	1.4
	H310A	1506	559	676	482	2.2	3.1	2.7
	I253A/H310A	1037	418	1037	412	1.0	2.5	2.5
^177^Lu-CHX-A" DTPA-	Wild type	5203	1709	833	1265	6.3	4.1	3.1
	H435A	4076	1235	835	932	4.9	4.4	3.3
	I253A	3514	1362	1189	1029	3.0	3.4	2.6
	H310A	2400	783	1104	774	2.2	3.1	3.1
	I253A/H310A	2699	453	1421	539	1.9	5.0	6.0
^131^I-	Wild type	2739	2085	560	659	4.9	4.2	1.3
	H435A	679	516	129	175	5.2	3.9	1.3
	I253A	568	516	141	159	4.0	3.6	1.1
	H310A	655	439	118	125	5.5	5.2	1.5
	I253A/H310A	602	422	107	120	5.6	5.0	1.4

^a^T/L, AUC_tumour_-to-AUC_liver_ ratio

^b^T/K, AUC_tumour_-to-AUC_kidney_ ratio

^c^T/B, AUC_tumour_-to-AUC_blood_ ratio

AUC ratios were also calculated for tumour-to-liver and tumour-to-kidney as these clearing organs could be dose limiting, especially for radiometal-labelled mutants. The lowest tumour-to-liver AUC ratios were observed with the ^90^Y-labelled (1) and ^177^Lu-labelled (1.9) hu3S193 I253A/H310A. Tumour-to-kidney ratios were lowest for ^90^Y-hu3S193 I253A (2.4) and ^90^Y-hu3S193 I253A/H310A (2.5) followed by ^90^Y-hu3S193 H310A (3.1) and ^177^Lu-hu3S193 H310A (3.1). Tumour-to-liver AUC ratios were superior for the ^131^I-labelled variants with the highest tumour-to-liver AUC ratio observed with ^131^I-hu3S193 I253A/H310A (5.6).

### Dosimetry calculation for radioimmunotherapy

**Fig. 2:**
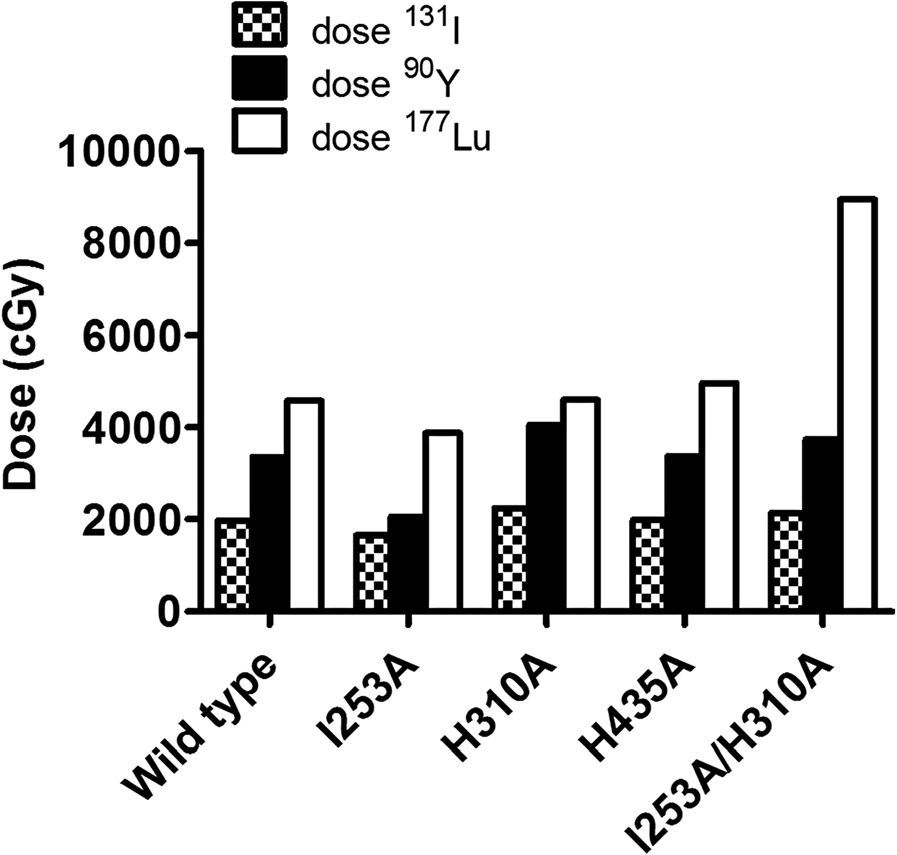
Maximum tumour dose predictions of ^131^I-, ^90^Y- and ^177^Lu-labelled hu3S193 wild type and variants. The limiting organ toxicity is set at 150 cGy for the bone marrow

To predict which hu3S193 mutant would be the best candidate for radioimmunotherapy, dose-limiting toxicity for the bone marrow was set at 150 cGy [21]. ^90^Y-CHX-A″ DTPA-hu3S193 H310A reached a tumour-absorbed dose 1.2-fold higher than hu3S193 wild type (H310A, 4050 cGy; wild type, 3300 cGy) (Fig. 2). ^177^Lu-CHX-A″ DTPA-hu3S193 I253A/H310A was predicted to be the best candidate for ^177^Lu-therapy, reaching a tumour dose of 8947 cGy compared to 4650 cGy for ^177^Lu-labelled wild type, limited by bone marrow toxicity (Fig. 2).

### Nano-SPECT imaging with ^177^Lu-CHX-A″ DTPA-labelled hu3S193 wild type and ^177^Lu-CHX-A″ DTPA-labelled hu3S193 I253A/H310A

Qualitative imaging was performed with hu3S193 wild type, hu3S193 I253A/H310A and huA33 control in A431 tumour-bearing mice. Mice were injected with 3.7 MBq (100 μCi, 270 μg) of radiolabelled antibody. HuA33 wild type was used as a negative control as the A431 cell line does not express A33 antigen. Quality controls showed that at day 0, only 3.4 % of ^177^Lu-CHX-A″ DTPA-labelled huA33 bound to A431 cells in vitro. In contrast, ^177^Lu-CHX-A″ DTPA-labelled hu3S193 wild type and I253A/H310A demonstrated a good binding at day 0 (72.6 ± 4.3 % and 53.9 ± 3.9 %, respectively; *n* = 3, SD) gradually decreasing to 25.0 ± 2.5 % (wild type) versus 18.4 ± 1.4 % (I253A/H310A) after 6 days of incubation in human serum at 37 °C.

**Fig. 3:**
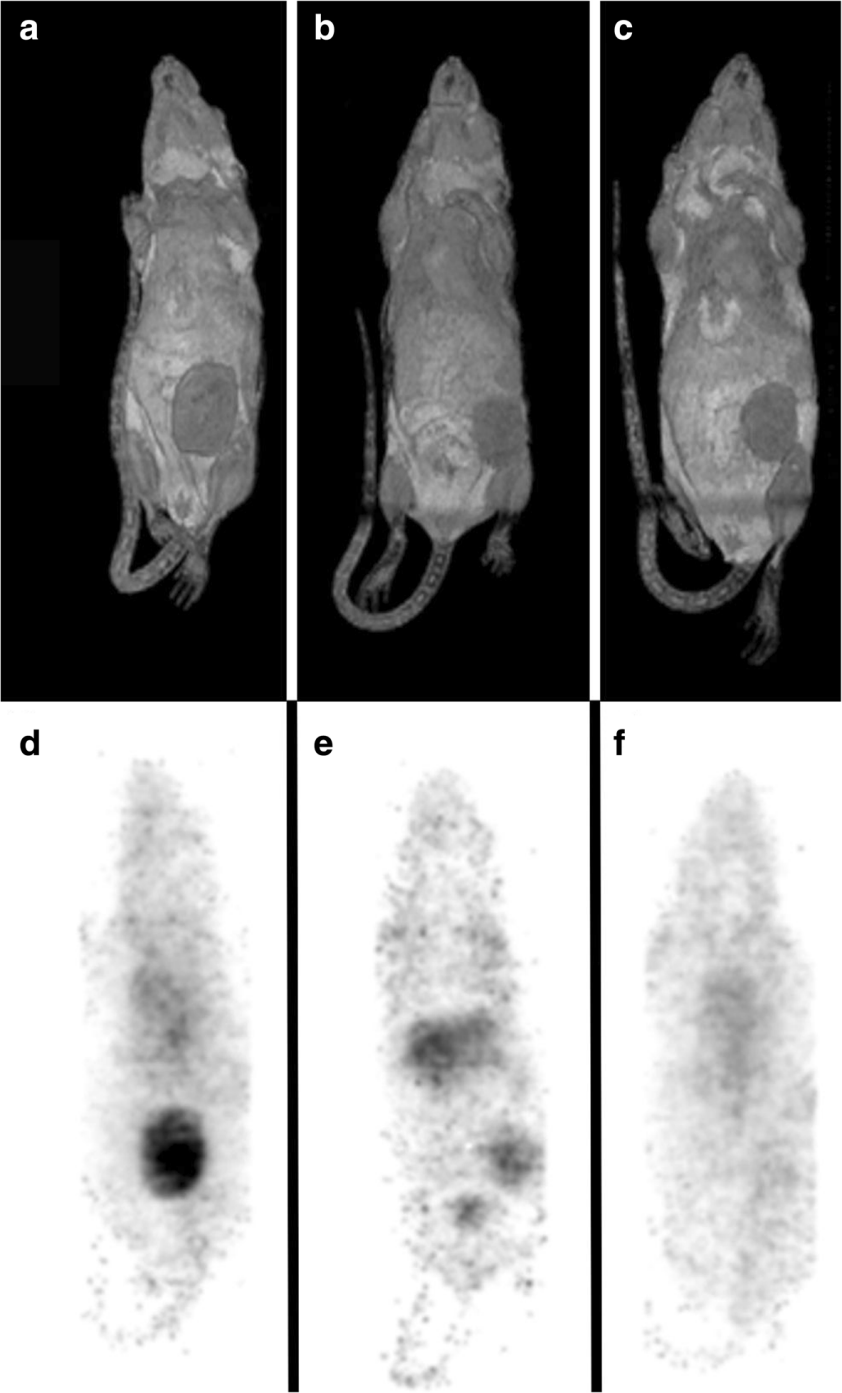
Nano-SPECT/MRI imaging with ^**177**^Lu-CHX-A″ DTPA-labelled hu3S193 wild type (**a**, **d**) and I253A/H310A mutant (**b**, **e**) and huA33 wild type antibody (**c**, **f**) at day 2 post injection in A431 tumour-bearing mice. Representative whole body surface-rendered MRI images (**a**–**c**) and maximum intensity projection SPECT image (**d**–**f**) are shown for each antibody. The T1 weighted MRI images clearly show the tumour (*grey solid mass*) surface of the lower abdomen, corresponding to the specific uptake of ^**177**^Lu-CHX-A″ DTPA-labelled hu3S193 wild type and I253A/H310A in tumours expressing Le^y^. Specific uptake of both hu3S193 wild type and hu3S193 I253A/H310A was demonstrated by the absence of huA33 uptake in the A33 antigen-negative A431 tumours

Whole body scans were obtained at 48 h post injection (Fig. 3). By 48 h, images showed tumour uptake for ^177^Lu-CHX-A″ DTPA-labelled hu3S193 wild type (Fig. 3a, d) and I253A/H310A (Fig. 3b, e). No tumour uptake was shown with the huA33 control demonstrating specific tumour uptake of hu3S193 wild type and I253A/H310A mutant in A431 tumours (Fig. 3c, 3f). High tumour uptake, high blood pool and background activity for hu3S193 wild type compared to hu3S193 I253A/H310A were observed. Volume of interest analysis for the heart, liver and tumour was used to calculate averaged uptake per pixel. Hu3S193 wild type had a lower tumour-to-heart ratio compared to I253A/H310A (wild type, 2.53 ± 0.07; I253A/H310A, 7.34 ± 2.05; *n* = 2). The tumour-to-liver ratio was higher for wild type (4.40 ± 1.51, *n* = 2) compared to I253A/H310A (1.35 ± 0.15; *n* = 2).

In a separate biodistribution study, mice were injected with ^177^Lu-CHX-A″ DTPA-labelled hu3S193 wild type or I253A/H310A (*n* = 5). After 48 h post injection, mice were sacrificed and organs were counted for radioactivity (Table 5). Biodistribution results confirmed reduced tumour activity of I253A/H310A (16.95 ± 3.60 %ID/g) compared to hu3S193 wild type (44.81 ± 3.35 %ID/g; *P* < 0.01). Tumour uptake of ^177^Lu-CHX-A″ DTPA-labelled I253A/H310A was specific and significantly higher than ^177^Lu-CHX-A″ DTPA-labelled huA33 (10.35 ± 0.54, *P* < 0.01). Importantly, tumour-to-blood ratios were 2.93 ± 0.65 (hu3S193 wild type), 0.49 ± 0.30 (huA33 control) and 16.56 ± 3.71 (hu3S193 I253A/H310A). Tumour-to-liver ratios were 7.71 ± 1.26 (hu3S193 wild type), 6.37 ± 0.62 (huA33 control) and 1.20 ± 0.23 (hu3S193 I253A/H310A).

**Table 5:** Biodistribution at 48 h post injection of ^177^Lu-CHX-A″ DTPA-labelled hu3S193 wild type and I253A/H310A in tumour-bearing mice

Tissue	Hu3S193 wild type	Hu3S193 I253A/H310A	HuA33
Tumour	44.81 ± 3.35	16.95 ± 3.60	10.35 ± 0.54
Blood	15.67 ± 2.47	1.05 ± 0.27	20.88 ± 1.25
Liver	5.99 ± 1.43	14.16 ± 1.28	6.37 ± 0.62
Spleen	9.32 ± 1.33	13.77 ± 2.57	9.60 ± 0.88
Kidney	8.27 ± 4.36	3.91 ± 0.41	7.98 ± 0.51
Lung	9.79 ± 1.55	1.95 ± 0.25	12.06 ± 0.99
Heart	5.72 ± 1.09	2.66 ± 0.16	6.03 ± 0.49
Muscle	2.01 ± 0.44	1.03 ± 0.08	2.34 ± 0.44

Higher protein doses were used for imaging with ^177^Lu-CHX-A″ DTPA-labelled hu3S193 antibodies (270 μg) and biodistribution (100 μg). Tumour uptake with 5 μg (~0.25 mg/kg) ^111^In-CHX-A″ DTPA-labelled hu3S193 antibodies was not significantly different from tumour uptake with 100 μg (~5 mg/kg) ^177^Lu-CHX-A″ DTPA-labelled hu3S193 antibodies as shown in Additional file 1: Figure S3, suggesting that at least at the 5 mg/kg dose level, no blocking of receptor has occurred.

## Discussion

Fc engineering is a promising technique to increase the therapeutic ratio for payload delivery. This approach aims to increase the therapeutic efficacy by reducing the terminal half-life of antibodies or Fc-containing antibody constructs, thus reducing the dose-limiting toxicity to the blood and bone marrow of intact radiolabelled therapeutic antibodies. In this study, four variants of an intact IgG1 antibody, hu3S193, with a broad range in half-life were created by site-directed mutagenesis and analysed in blood clearance studies and biodistribution studies. Our results show that hu3S193 can be engineered with improved therapeutic ratios for ^90^Y- and ^177^Lu-based therapy, with the best candidate being hu3S193 I253A/H310A for ^177^Lu-based therapy, achieving almost double tumour-to-blood AUC ratios compared to wild-type hu3S193.

Four Fc variants of an intact IgG1 antibody, hu3S193, with a broad range in serum half-life were created by site-directed mutagenesis and analysed in preclinical blood clearance studies and biodistribution studies. Non-specific uptake of hu3S193 wild type in a Lewis-Y-negative cell line SW1222 is known to be low [26]; in addition, in this study, a non-specific IgG1 control antibody huA33 showed low uptake in A431 tumours at 72 h post injection. Therefore, the tumour uptake of radiolabelled hu3S193 mutants was specific and antigen-mediated.

Early engineering approaches using deletion of constant domains to reduce the serum half-life of antibodies have shown that tumour-to-normal dose ratios can be improved [1, 10, 11, 37]. More recently, studies by Kenanova et al. reported on therapy prospects of radioiodinated and radiometal-labelled Fc-engineered anti-carcinoembryonic antigen (CEA) scFv-Fc fragments [20, 21]. Dosimetry calculations predicted that the ^131^I-labelled scFv-Fc H310A/H435Q could deliver >7000 cGy, limited by red marrow toxicity. ^90^Y-labelled scFv-Fc I253A was best suited for ^90^Y delivery, although dose predictions did not match the intact cT84.66 antibody. The current study extends their findings to an intact IgG1 and includes predictions for ^177^Lu-based therapy. In contrast to the anti-CEA study by Kenanova et al., the radioiodinated anti-Le^y^ hu3S193 variants cleared much faster than the radioiodinated scFv-Fc fragments, and therefore, no therapeutic benefit could be obtained from the radioiodinated hu3S193 variants. The hu3S193 variants might be more sensitive to deiodination in vivo compared to the scFv-Fc variants. To study the benefit for RIT of using radioiodinated hu3S193 variants with faster blood clearance, other radioiodination methods such as site-specific radioiodination of cysteine-containing hu3S193 variants could be explored [38].

Whilst a doubling in dose to the maximum predicted dose calculated for hu3S193 wild type was predicted and a doubling in the tumour-to-blood AUC ratios achieved, the dose needed for tumour eradication in patients is likely to be higher than that achievable with a single infusion. Le^y^-expressing tumours such as colon, lung, ovarian and breast cancer require radiation doses of at least 60 Gy to eradicate solid tumours in patients [1]. Reducing the half-life of systemically administered antibodies is usually associated with reduced uptake in tumour; however, AUC ratios of tumour and blood address the varying effects of alterations in antibody kinetics, and our results confirm that selective IgG1 mutations do provide benefit in therapeutic ratios. Approaches to further increase therapeutic efficacy of Fc-engineered intact antibodies with reduced half-lives can include dose fractionation [39], use in pretargeting radioimmunotherapy approaches [40] or combination with chemotherapy [28, 29]. To date, pretargeting approaches show advantages compared to improvements in one-step RIT for improved tumour-to-normal tissue AUC ratios and tumour responses in preclinical models of solid tumours [1], but challenges remain to develop non-immunogenic and translatable approaches for human trials.

Due to the toxicity limitations of RIT and patient tumour variability, it is useful to develop theranostics to guide dosimetry in patients, exemplified in this study by ^177^Lu-labelled I253A/H310A. The imaging results reflect the biodistribution data; the highest tumour uptake was seen with hu3S193 wild type, but specific tumour uptake was still achievable with the fastest clearing hu3S193 variant I253A/H310A, and tumour-to-blood ratios were far higher at earlier time points with the mutant hu3S193 constructs. However, because ^177^Lu can only be imaged using SPECT cameras, a PET equivalent such as ^89^Zr-labelled I253A/H310A might be a better alternative and may allow better quantification of tumour uptake and saturability in vivo. We are currently evaluating ^89^Zr-labelled hu3S193 variants in tumour-bearing mice in small animal PET studies.

A higher protein dose was used for ^177^Lu-imaging (270 μg) and biodistribution (100 μg) studies versus ^111^In-biodistribution (5 μg) studies. We demonstrated that tumour uptake at 5 and 100 μg protein doses were comparable. Lewis-Y expression is very high on A431 cells and similar to MCF-7 cells, estimated to express around 10^6^–10^7^ antibody binding sites [26]. A recent report on ABT-806, a humanised antibody that specifically targets epidermal growth factor receptor (EGFR), reports on cold competition blocking of the 806 antibody in A431 tumours [41]. EGFR is highly expressed in A431 cells, and therefore, more than 20 mg/kg of cold ABT-806 antibody was needed to allow competition in vivo. It is therefore expected that competition with hu3S193 antibodies will only occur at doses higher than 20 mg/kg, which aligns with the observation that at the 5 mg/kg dose (100 μg), no reduction in tumour uptake was observed at 48 h post injection.

Although significant increases in therapeutic doses were predicted with some variants compared to hu3S193 wild type, increased toxicities to normal tissue such as the liver and kidneys were also predicted. A shift in toxicity from the bone marrow to liver as toxicity limiting organ for radiometal-labelled antibody variants with a faster blood clearance is not surprising. As the antibody variants clear faster from the body, radioactivity exposure to the blood is reduced. In addition, due to the reduced binding affinity of the variants for FcRn, an increase in catabolism is expected as the variants are no longer recycled in the blood circulation and degraded in the lysosomes (e.g. hu3S193 I253A/H310A) [19]. In contrast to radioiodine, the small radiometal-labelled metabolites are trapped in the lysosomes of hepatocytes [42], and thus, a higher initial uptake of radioactivity observed in the liver is the result of enhanced accumulation of radiometal chelates after degradation of the variants in the lysosomes of hepatocytes. However, a mouse model might not be readily translatable to human studies to estimate liver and kidney toxicities developing from catabolites due to differences in stability of radiolabelled conjugates in human serum and relative size of tissue compartments between mouse and human. A theranostic might therefore also aid in appropriate dose selection when using engineered intact antibodies with altered pharmacokinetics.

## Conclusions

Four Fc variants of an intact IgG1 antibody, hu3S193, with a broad range in serum half-life were created by site-directed mutagenesis and analysed in preclinical blood clearance studies and biodistribution studies. We have shown that hu3S193 can be engineered with improved therapeutic ratios for ^90^Y- and ^177^Lu-based therapy, with the best candidate being hu3S193 I253A/H310A for ^177^Lu-based therapy. These results are highly relevant to utilising Fc engineering as a tool to improve the clinical application of hu3S193 and other intact antibodies especially for payload delivery.

## Additional file

Additional file 1: Figures S1-S3; Tables S1-S3. (DOC 1691 kb)
