# Supplementary material for: Engineering anti-Lewis-Y hu3S193 antibodies with improved therapeutic ratio for radioimmunotherapy of epithelial cancers
Source: EJNMMI Res. 2016 Mar 17;6:26. doi: 10.1186/s13550-016-0180-0 (PMC4796444; doi:10.1186/s13550-016-0180-0)
Supplement: Additional file 1: — Figures S1-S3; Tables S1-S3. (DOC 1691 kb) [file 13550_2016_180_MOESM1_ESM.doc]

**Additional file 1**

**Engineering Anti-Lewis-Y Hu3S193 Antibodies with Improved Therapeutic Ratio for Radioimmunotherapy of Epithelial Cancers**

EJNMMI Research

Ingrid J.G. Burvenich^1,2^, Fook-Thean Lee^1^, Graeme J. O’Keefe^2,3^, Dahna Makris^1^, Diana Cao^1^, Sylvia Gong^3^, Angela Rigopoulos^1^, Laura C. Allan^1^, Martin W. Brechbiel^4^, Zhanqi Liu^1^, Paul A. Ramsland^5,6,7,8^, and Andrew M. Scott^1,2,3,9^

**Authors and affiliations.**

^1^Tumour Targeting Laboratory, Ludwig Institute for Cancer Research and Olivia Newton-John Cancer Research Institute, Melbourne, VIC, Australia

^2^School of Cancer Medicine, La Trobe University, Melbourne, VIC, Australia

^3^Department of Molecular Imaging and Therapy, Austin Health, Melbourne, Australia

^4^Radioimmune & Inorganic Chemistry Section, Radiation Oncology Branch, Center for Cancer Research, National Cancer Institute, Bethesda, MD, USA

^5^School of Science, RMIT University, Bundoora, VIC, Australia

^6^Centre for Biomedical Research, Burnet Institute, Melbourne, VIC, Australia

^7^Department of Immunology, Monash University, Melbourne, VIC, Australia

^8^Department of Surgery Austin Health, University of Melbourne, Heidelberg, VIC, Australia

^9^Faculty of Medicine, University of Melbourne, Melbourne, VIC, Australia

**To whom requests for reprints should be addressed:** Andrew M. Scott, Olivia Newton-John Cancer Research Institute, 145-163 Studley Road, Heidelberg, VIC 3084, Australia. Phone: +61 3 9496 5876, Fax: +61 9496 5334; E-mail: [andrew.scott@onjcri.org.au](mailto:andrew.scott@onjcri.org.au)


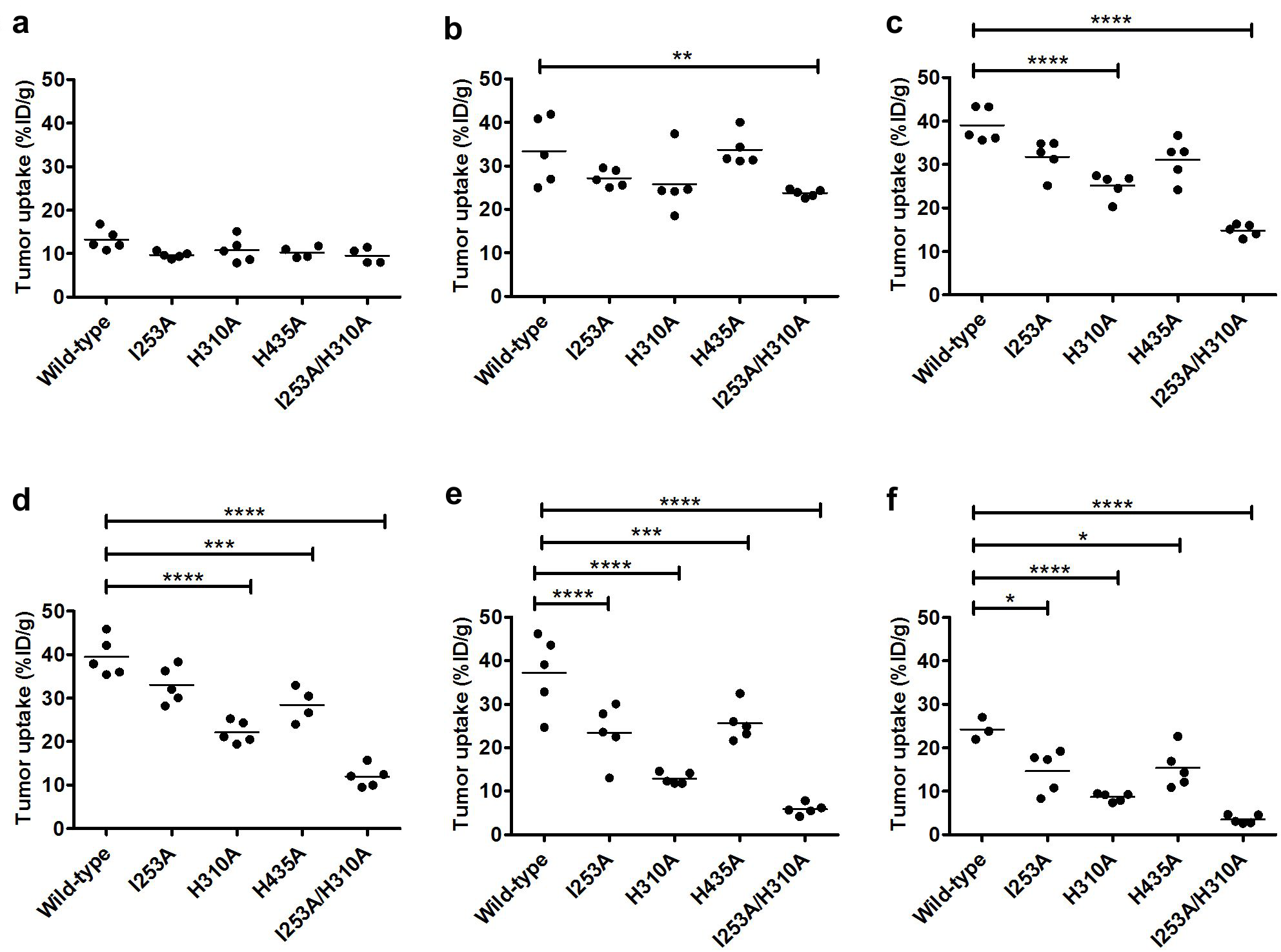


**Supplementary Fig. 1** Two-way ANOVA analysis (Bonferroni post-test) of tumour uptake with ^111^In-CHX-A″ DTPA-labelled hu3S193 antibodies variants compared to wild-type at 4 (**a**), 24 (**b**), 48 (**c**), 72 (**d**), 120 (**e**) and 168 (**f**) hours post injection. *, *P* < 0.05; **, *P* < 0.01, ***, *P* < 0.001; ****, *P* < 0.0001


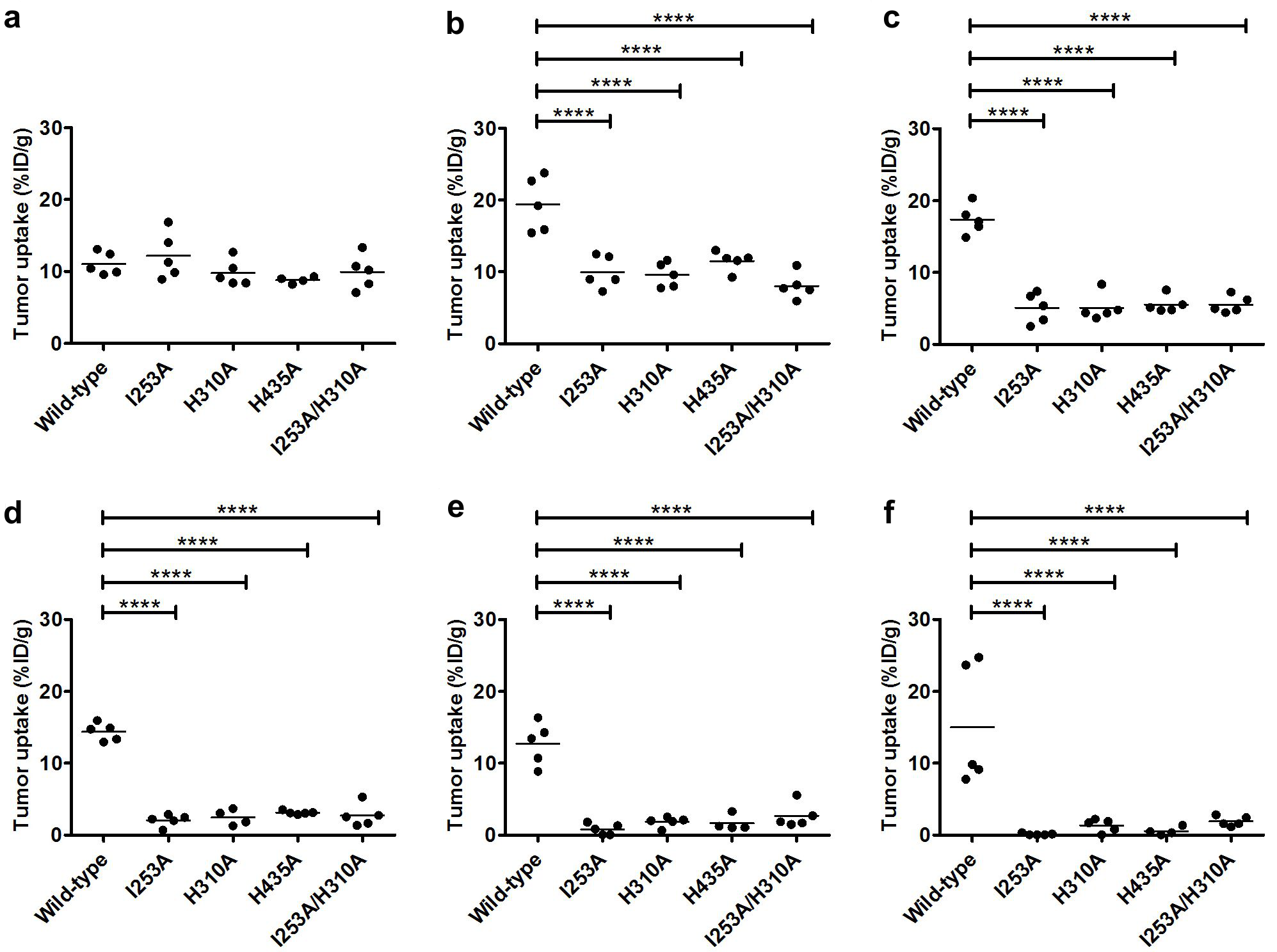


**Supplementary Fig. 2** Two-way ANOVA analysis (Bonferroni post-test) of tumour uptake with ^131^I-labelled hu3S193 antibodies variants compared to wild-type at 4 (**a**), 24 (**b**), 48 (**c**), 72 (**d**), 120 (**e**) and 168 (**f**) hours post injection. ****, *P* < 0.0001


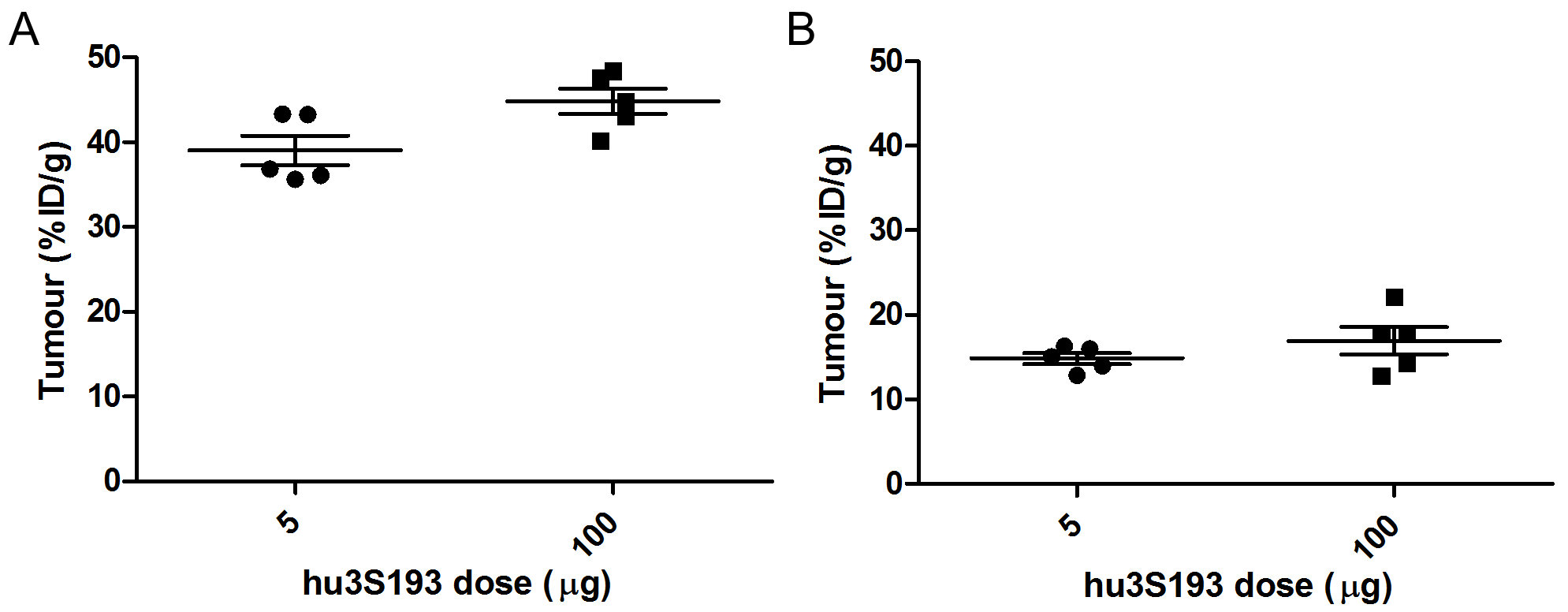


**Supplementary Fig. 3** Comparison of tumour uptake at different dose levels of (A) wild-type hu3S193 and (B) I253A/H310A at 48 hours post injection.

Supplementary Table 1 *In vitro* binding characteristics of hu3S193 wild-type and mutant antibodies to Le^Y^-BSA (BIAcore) and Le^y^-expressing A431 cells (FACS)

| Antibody | BIAcore LewisY-BSA | | | FACS A431 cells |
| --- | --- | --- | --- | --- |
|  | k_on_  (× 10^5^/Ms) | k_off_  (×10^-2^/s) | K_D_  (10^-9^ mol/L) | Mean Yellow Fluorescence |
| wild-type | 2.44 | 3.38 | 115 | 4640 |
| I253A | 1.75 | 6.72 | 118 | 4181 |
| H310A | 1.46 | 3.97 | 126 | 4180 |
| H435A | 1.62 | 5.20 | 111 | 4185 |
| I253A/H310A | 3.0 | 3.77 | 118 | 4445 |
|  | | | | |

**Supplementary Table 2** Biodistribution of ^111^In-CHX-A″ DTPA-labelled hu3S193 wild-type and variants in A431 tumour-bearing mice

| Organ | Time (hours) | | | | | |
| --- | --- | --- | --- | --- | --- | --- |
|  | 4 | 24 | 48 | 72 | 120 | 168 |
| ^111^In-CHX-A″ DTPA hu3S193 I253A | | | | | | |
| Blood | 38.74 ± 3.57*  15.52 ± 3.45  10.38 ± 0.62  12.80 ± 1.00  15.59 ± 2.12  9.64 ± 0.74  4.55 ± 0.70 | 17.99 ± 1.57  10.16 ± 1.74  9.32 ± 1.56  9.00 ± 0.91  8.94 ± 1.28  27.17 ± 2.00  3.96 ± 0.53 | 10.19 ± 1.04  8.75 ± 1.69  8.03 ± 1.70  7.01 ± 0.93  5.91 ± 0.83  31.75 ± 3.98  3.27 ± 0.59 | 7.64 ± 1.32  7.15 ± 0.32  6.01 ± 0.71  6.20 ± 0.19  4.68 ± 0.73  33.11 ± 4.05  2.66 ± 0.20 | 2.99 ± 0.73  5.61 ± 1.09  5.86 ± 0.52  5.09 ± 0.82  2.58 ± 0.57  23.68 ± 6.52  2.17 ± 0.56 | 1.14 ± 0.63  4.23 ± 0.51  2.77 ± 0.76  3.65 ± 1.31  1.29 ± 0.37  14.66 ± 4.80  1.43 ± 0.32 |
| Liver |  |  |  |  |  |  |
| Spleen |  |  |  |  |  |  |
| Kidney |  |  |  |  |  |  |
| Lung |  |  |  |  |  |  |
| Tumour |  |  |  |  |  |  |
| Femur |  |  |  |  |  |  |
| ^111^In-CHX-A″ DTPA hu3S193 H310A | | | | | | |
| Blood | 30.52 ± 5.06  11.85 ± 2.09  7.47 ± 1.43  9.96 ± 1.40  13.35 ± 2.81  10.79 ± 2.87  3.55 ± 0.44 | 11.05 ± 1.62  11.19 ± 2.41  7.30 ± 1.16  7.13 ± 0.98  5.97 ± 0.75  22.85 ± 2.93  3.22 ± 0.63 | 5.61 ± 2.01  7.96 ± 1.06  6.85 ± 1.55  6.11 ± 0.94  3.54 ± 0.89  25.10 ± 2.91  2.50 ± 0.58 | 2.56 ± 0.60  7.32 ± 0.41  4.46 ± 1.04  4.76 ± 0.27  2.35 ± 0.26  22.12 ± 2.55  1.95 ± 0.23 | 0.54 ± 0.16  4.84 ± 0.35  4.10 ± 0.58  3.58 ± 0.42  1.07 ± 0.16  12.89 ± 1.33  1.25 ± 0.28 | 0.17 ± 0.06  3.84 ± 0.26  3.30 ± 0.84  2.88 ± 0.39  0.82 ± 0.14  8.64 ± 0.96  1.29 ± 0.25 |
| Liver |  |  |  |  |  |  |
| Spleen |  |  |  |  |  |  |
| Kidney |  |  |  |  |  |  |
| Lung |  |  |  |  |  |  |
| Tumour |  |  |  |  |  |  |
| Femur |  |  |  |  |  |  |
| ^111^In-CHX-A″ DTPA hu3S193 H435A | | | | | | |
| Blood | 37.97 ± 2.68  9.49 ± 0.76  7.39 ± 0.41  11.53 ± 0.36  15.04 ± 1.47  10.25 ± 1.30  3.72 ± 0.60 | 17.09 ± 1.59  7.19 ± 0.78  7.23 ± 1.04  8.45 ± 1.40  8.53 ± 1.14  33.69 ± 3.77  3.31 ± 0.49 | 7.65 ± 1.60  6.08 ± 1.30  5.63 ± 1.09  5.47 ± 0.74  5.23 ± 1.33  31.10 ± 4.76  2.20 ± 0.41 | 5.18 ± 1.40  5.80 ± 2.11  6.48 ± 0.45  5.08 ± 0.58  2.82 ± 0.68  28.48 ± 4.00  1.78 ± 0.41 | 3.19 ± 0.62  3.82 ± 0.57  3.65 ± 0.70  4.34 ± 0.48  2.36 ± 0.27  25.60 ± 4.16  1.41 ± 0.08 | 1.16 ± 0.62  3.21 ± 1.11  2.66 ± 0.49  3.07 ± 0.46  1.25 ± 0.31  15.34 ± 4.65  1.12 ± 0.19 |
| Liver |  |  |  |  |  |  |
| Spleen |  |  |  |  |  |  |
| Kidney |  |  |  |  |  |  |
| Lung |  |  |  |  |  |  |
| Tumour |  |  |  |  |  |  |
| Femur |  |  |  |  |  |  |
| ^111^In-CHX-A″ DTPA hu3S193 I253A/H310A | | | | | | |
| Blood | 28.84 ± 6.05  15.34 ± 3.19  9.54 ± 2.46  10.40 ± 2.35  11.33 ± 2.66  8.92 ± 2.00  3.28 ± 0.77 | 5.28 ± 0.81  18.68 ± 1.34  11.26 ± 3.24  6.30 ± 0.70  3.88 ± 0.47  23.72 ± 0.85  3.42 ± 0.61 | 0.46 ± 0.07  12.41 ± 0.85  7.92 ± 1.56  4.04 ± 0.14  1.63 ± 1.23  14.84 ± 1.43  2.03 ± 0.14 | 0.08 ± 0.03  10.42 ± 2.89  7.43 ± 2.84  3.50 ± 0.97  1.18 ± 0.23  11.92 ± 2.46  1.66 ± 0.13 | 0.00 ± 0.00  5.96 ± 0.92  4.28 ± 1.79  2.47 ± 0.40  0.65 ± 0.11  5.85 ± 1.31  1.21 ± 0.26 | 0.00 ± 0.00  5.03 ± 0.61  4.13 ± 0.80  2.16 ± 0.37  0.46 ± 0.09  3.52 ± 1.00  1.04 ± 0.13 |
| Liver |  |  |  |  |  |  |
| Spleen |  |  |  |  |  |  |
| Kidney |  |  |  |  |  |  |
| Lung |  |  |  |  |  |  |
| Tumour |  |  |  |  |  |  |
| Femur |  |  |  |  |  |  |
| ^111^In-CHX-A″ DTPA wild-type | | | | | | |
| Blood | 35.56 ± 5.48  10.65 ± 2.02  8.55 ± 1.32  11.10 ± 1.38  16.23 ± 3.06  13.17 ± 2.40  4.23 ± 1.13 | 21.83 ± 3.32  7.11 ± 1.38  6.60 ± 1.54  9.34 ± 1.66  11.00 ± 2.96  33.42 ± 7.75  3.75 ± 0.71 | 14.50 ± 1.11  5.52 ± 0.74  5.50 ± 0.74  7.59 ± 0.81  7.64 ± 1.39  39.01 ± 3.92  3.01 ± 0.30 | 9.97 ± 1.85  4.29 ± 0.91  3.97 ± 1.74  6.01 ± 0.79  5.57 ± 1.31  39.27 ± 4.33  2.55 ± 0.26 | 5.78 ± 2.12  3.18 ± 0.82  3.51 ± 0.80  5.28 ± 1.17  3.56 ± 1.26  37.26 ± 8.67  1.97 ± 0.60 | 1.06 ± 0.32  2.91 ± 0.49  3.14 ± 0.30  3.68 ± 0.37  2.20 ± 0.35  24.24 ± 2.57  1.75 ± 0.16 |
| Liver |  |  |  |  |  |  |
| Spleen |  |  |  |  |  |  |
| Kidney |  |  |  |  |  |  |
| Lung |  |  |  |  |  |  |
| Tumour |  |  |  |  |  |  |
| Femur |  |  |  |  |  |  |
|  |  |  |  |  |  |  |
| *Data presented as percentage injected dose per gram tissue (mean ± SD) | | | | | | |

**Supplementary Table 3** Biodistribution of ^125^I-labelled hu3S193 wild-type and ^131^I-labelled mutants in A431 tumour-bearing mice

| Organ  (%ID/g) | Time (hours) | | | | | |
| --- | --- | --- | --- | --- | --- | --- |
|  | 4 | 24 | 48 | 72 | 120 | 168 |
| ^131^I-hu3S193 I253A | | | | | | |
| Blood | 29.95 ± 3.59 | 6.94 ± 1.34 | 1.09 ± 0.32 | 0.04 ± 0.08 | 0.00 ± 0.00 | 0.00 ± 0.00 |
| Liver | 7.92 ± 0.85 | 1.82 ± 0.40 | 0.40 ± 0.07 | 0.11 ± 0.03 | 0.00 ± 0.00 | 0.00 ± 0.00 |
| Spleen | 7.27 ± 1.71 | 1.61 ± 0.47 | 0.32 ± 0.08 | 0.05 ± 0.02 | 0.00 ± 0.00 | 0.00 ± 0.00 |
| Kidney | 8.86 ± 1.23 | 2.08 ± 0.46 | 0.39 ± 0.08 | 0.03 ± 0.04 | 0.00 ± 0.00 | 0.00 ± 0.00 |
| Lung | 12.58 ± 2.29 | 3.82 ± 0.98 | 0.65 ± 0.17 | 0.03 ± 0.05 | 0.00 ± 0.00 | 0.00 ± 0.00 |
| Tumour | 10.01 ± 3.25 | 9.95 ± 2.26 | 5.07 ± 2.10 | 2.03 ± 0.81 | 0.78 ± 0.79 | 0.10 ± 0.14 |
| Femur | 3.59 ± 0.78 | 0.96 ± 0.30 | 0.20 ± 0.05 | 0.03 ± 0.03 | 0.00 ± 0.00 | 0.00 ± 0.00 |
| ^131^I-hu3S193 H310A | | | | | | |
| Blood | 32.56 ± 3.00 | 3.85 ± 0.35 | 0.01 ± 0.02 | 0.00 ± 0.00 | 0.00 ± 0.00 | 0.00 ± 0.00 |
| Liver | 8.26 ± 0.67 | 1.10 ± 0.07 | 0.08 ± 0.02 | 0.00 ± 0.00 | 0.00 ± 0.00 | 0.00 ± 0.00 |
| Spleen | 6.52 ± 0.86 | 0.74 ± 0.06 | 0.06 ± 0.04 | 0.00 ± 0.00 | 0.00 ± 0.00 | 0.00 ± 0.00 |
| Kidney | 8.65 ± 0.96 | 1.20 ± 0.08 | 0.06 ± 0.02 | 0.00 ± 0.00 | 0.00 ± 0.00 | 0.00 ± 0.00 |
| Lung | 13.45 ± 2.76 | 1.08 ± 0.15 | 0.08 ± 0.04 | 0.00 ± 0.00 | 0.00 ± 0.00 | 0.00 ± 0.00 |
| Tumour | 9.80 ± 1.83 | 9.58 ± 1.73 | 5.10 ± 1.87 | 2.43 ± 1.10 | 1.81 ± 0.71 | 1.45 ± 0.99 |
| Femur | 3.23 ± 0.21 | 0.61 ± 0.07 | 0.05 ± 0.01 | 0.00 ± 0.00 | 0.00 ± 0.00 | 0.00 ± 0.00 |
| ^131^I-hu3S193 H435 | | | | | | |
| Blood | 32.46 ± 2.63 | 8.46 ± 1.89 | 2.27 ± 1.21 | 0.69 ± 0.17 | 0.00 ± 0.00 | 0.00 ± 0.00 |
| Liver | 6.30 ± 0.87 | 1.79 ± 0.39 | 0.59 ± 0.32 | 0.22 ± 0.04 | 0.00 ± 0.00 | 0.00 ± 0.00 |
| Spleen | 5.57 ± 0.90 | 1.34 ± 0.34 | 0.59 ± 0.31 | 0.15 ± 0.04 | 0.00 ± 0.00 | 0.00 ± 0.00 |
| Kidney | 8.86 ± 0.96 | 2.40 ± 0.56 | 0.70 ± 0.36 | 0.26 ± 0.06 | 0.00 ± 0.00 | 0.00 ± 0.00 |
| Lung | 13.89 ± 1.84 | 3.92 ± 0.74 | 1.23 ± 0.75 | 0.40 ± 0.09 | 0.00 ± 0.00 | 0.00 ± 0.00 |
| Tumour | 8.79 ± 0.45 | 11.52 ± 1.38 | 5.53 ± 1.18 | 3.18 ± 0.22 | 1.64 ± 1.09 | 0.54 ± 0.58 |
| Femur | 2.94 ± 0.36 | 0.99 ± 0.20 | 0.30 ± 0.12 | 0.13 ± 0.03 | 0.01 ± 0.02 | 0.00 ± 0.00 |
| ^131^I-hu3S193 I253A/H310A | | | | | | |
| Blood | 30.20 ± 3.97 | 3.88 ± 1.32 | 0.06 ± 0.09 | 0.00 ± 0.00 | 0.00 ± 0.00 | 0.00 ± 0.00 |
| Liver | 7.36 ± 1.09 | 1.02 ± 0.20 | 0.07 ± 0.07 | 0.00 ± 0.00 | 0.00 ± 0.00 | 0.00 ± 0.00 |
| Spleen | 4.43 ± 1.00 | 0.83 ± 0.19 | 0.07 ± 0.05 | 0.00 ± 0.00 | 0.00 ± 0.00 | 0.00 ± 0.00 |
| Kidney | 8.26 ± 0.94 | 1.17 ± 0.38 | 0.04 ± 0.05 | 0.00 ± 0.00 | 0.00 ± 0.00 | 0.00 ± 0.00 |
| Lung | 11.53 ± 1.39 | 1.98 ± 0.72 | 0.11 ± 0.11 | 0.00 ± 0.00 | 0.00 ± 0.00 | 0.00 ± 0.00 |
| Tumour | 9.90 ± 2.42 | 8.04 ± 1.82 | 5.10 ± 0.76 | 3.04 ± 1.58 | 1.92 ± 0.53 | 1.43 ± 0.27 |
| Femur | 2.61 ± 0.26 | 0.46 ± 0.14 | 0.04 ± 0.03 | 0.04 ± 0.07 | 0.00 ± 0.00 | 0.00 ± 0.00 |
| ^125^I-hu3S193 wild-type | | | | | | |
| Blood | 39.37 ± 3.33 | 22.17 ± 1.81 | 15.09 ± 1.70 | 13.18 ± 1.91  3.06 ± 0.25  2.95 ± 0.42  3.98 ± 0.45  6.86 ± 1.38 | 7.13 ± 1.46  2.11 ± 0.41  2.27 ± 0.54  2.45 ± 0.46 | 4.44 ± 1.56  0.92 ± 0.35  0.75 ± 0.23  1.29 ± 0.49  1.25 ± 0.68 |
| Liver | 12.44 ± 2.23 | 5.50 ± 1.01 | 4.16 ± 0.91  4.43 ± 1.26  4.76 ± 0.59  8.28 ± 1.35 |  |  |  |
| Spleen | 10.19 ± 2.54 | 5.91 ± 1.16 |  |  |  |  |
| Kidney | 13.03 ± 1.26 | 6.85 ± 0.71 |  |  |  |  |
| Lung | 18.16 ± 2.49 | 11.51 ± 1.54 |  |  | 4.23 ± 1.19 |  |
| Tumour | 9.02 ± 0.73  4.48 ± 0.75 | 17.37 ± 1.25  3.25 ± 0.40 | 17.58 ± 2.60  2.44 ± 0.43 | 17.76 ± 1.72  1.85 ± 0.16 | 15.10 ± 3.89  1.02 ± 0.32 | 11.40 ± 4.56  0.68 ± 0.20 |
| Femur |  |  |  |  |  |  |
|  |  |  |  |  |  |  |
| *Data presented as percentage injected dose per gram tissue (mean ± SD) | | | | | | |
